# Acute Enterovirus Encephalitis as a Cause for Isolated Visual and Auditory Hallucinations in a 22-Year-Old Patient

**DOI:** 10.1155/2021/6687735

**Published:** 2021-04-23

**Authors:** M. Deest, C. Eberlein, S. Bleich, H. Frieling, T. Skripuletz, A. Neyazi

**Affiliations:** ^1^Department of Psychiatry, Social Psychiatry and Psychotherapy, Hannover Medical School, Carl-Neuberg-Straße 1, 30625 Hannover, Germany; ^2^Department of Neurology, Hannover Medical School, Carl-Neuberg-Straße 1, 30625 Hannover, Germany

## Abstract

Viral encephalitis often presents with severe illness, headache, fever, behavioral changes, altered level of consciousness, and focal neurologic deficits. One of the most feared kind of virus encephalitis is herpes simplex encephalitis; however, other central virus infections are also capable of presenting with psychiatric symptoms. Here, we report the case of a 22-year-old woman with first time visual and auditory hallucinations due to an acute enterovirus encephalitis with no cerebrospinal fluid abnormalities but a positive PCR result for enterovirus (ECHO). During treatment, the symptoms deteriorated, and she hat to be shifted to the sheltered ward because of imperative suicidal auditory hallucinations. Under treatment with risperidone and olanzapine, symptoms suddenly stopped and did not reoccur under subsequent reduction of the antipsychotic medication.

## 1. Introduction

Viral encephalitis often presents with severe illness, headache, fever, behavioral changes, altered level of consciousness, and focal neurologic deficits. Over 100 viruses have been linked with acute infections of the central nervous system [1]. One of the most feared kind of virus encephalitis is the herpes simplex encephalitis leading to death in up to 70% when untreated [2]. However, other central virus infections are also capable of presenting with psychiatric symptoms but are often neglected in everyday clinical practice [2]. We report the case of a 22-year-old woman with first time visual and auditory hallucinations due to an acute enterovirus encephalitis with no cerebrospinal fluid (CSF) abnormalities but a positive PCR result for enteroviruses.

## 2. Case Presentation

In January 2018, a 22-year-old female patient from Madagascar, living in Germany since two years, introduced herself at our emergency ward of the Department of Psychiatry and Psychotherapy. She was suffering from acute auditory hallucinations since seven days and acute visual hallucinations beginning at the day of admission. As per her narration, she was hearing the same voice over and over, telling her to “go to the sea” and seeing an old, faceless woman, which would be the likely source of the voice. She never had this kind of symptoms before in her life. There were no abnormalities of consciousness or orientation and no hints for other psychopathology changes, except that she was very frightened by the hallucinations. She reported episodic severe headache which would intensify the hallucinations. One week before onset of psychiatric symptoms, the patient suffered from severe diarrhea for several days. She was working as an elderly care nurse in a retirement home where many patients suffered from diarrhea simultaneously. The internal and neurological examinations were unremarkable, particularly no fever or neck stiffness was observed. The laboratory examinations were also within normal range, especially no elevated levels of C-reactive protein or lymphocytosis were found. Cranial MRI and an EEG were unremarkable. CSF analysis showed a normal cell count (1.0 cells/*μ*l) and a normal blood-CSF-barrier function (albumin ratio 6.64, total protein 474 mg/l). There was no intrathecal synthesis of IgG, IgA, or IgM and no CSF-specific oligoclonal bands were detected (type 1). Testing of antineuronal and autoimmune encephalitis antibodies in the serum and CSF showed no pathologies. PCRs and the antibody-specific indices for CMV, HSV, EBV, and VZV were negative. However, PCR for enteroviruses (ECHO) was positive, indicating an acute/subacute infection. The patients' stool was not tested because she did not show any abdominal symptoms anymore.

A symptomatic antipsychotic and anxiolytic therapy with risperidone (3 mg/day) and lorazepam (5.5 mg/day) and an analgesic therapy with NSAIDs were initiated. However, the symptoms of the patient got more severe. She reported that the quality of the auditory hallucinations changed and that the voice was telling her to leave and commit suicide. Olanzapine (7.5 mg/day) was added as a second antipsychotic drug; however, the symptoms worsened, and she had to be to shifted to the sheltered ward of our department as she reported increasing imperative, suicidal auditory hallucinations. 28 days after the first occurrence of the symptoms, the hallucinations and the headache suddenly stopped within a day under the above-mentioned treatment. In the next days, the medication was changed to aripiprazole (10 mg/day) because of an increased serum prolactin level with reported lactation. On subsequent reduction of olanzapine, no hallucinations or other symptoms recurred, and the patient could be discharged from the hospital treatment with a medication of aripiprazole (10 mg/day) and olanzapine (2.5 mg/day). 4 months after discharge, the patient was free of symptoms under treatment with 10 mg of aripiprazole.

## 3. Discussion

One of the most important differential diagnosis of acute psychosis is the herpes simplex encephalitis leading to death in up to 70% of cases when untreated [1]. However, other central nervous system virus infections causing encephalitis are also capable of presenting with psychiatric symptoms but are often neglected in clinical everyday life [2]. Among the infectious diseases of the central nervous system, enterovirus infections are the most common and account for up to 77% of all viral meningitis cases in adults [3] which typically leads to headache, fever, and neck stiffness. However, enteroviruses also can cause an encephalitis which typically results in an altered mental status and focal neurological signs [4]. Enterovirus-induced encephalitis is normally diagnosed via inflammatory changes in the CSF, e.g., pleocytosis, and detection of the viral RNA via PCR in the CSF [5]. In our case, apart from the hallucinations, the patient was only suffering from uncharacteristic headache and had no fever. Furthermore, there were no inflammatory changes, but PCR detected enterovirus RNA in the CSF.

We recently showed that in up to 15% of all cases with enterovirus-induced meningitis/encephalitis, a normal CSF cell count occurs. Furthermore, enterovirus encephalitis can cause isolated cranial nerve involvement [6]. Other case reports also showed that the clinical manifestations of a central enterovirus infection are within a broad range. Exemplary, a case with behavioral changes and autistic features such as impairment of communication, mutism, and lack of eye contact was reported [7]. Another case report described a recurrent limbic encephalitis with convulsions, abnormal behavior, and consciousness disturbance due to an enterovirus infection [8].

Regarding possible differential diagnoses, one should also consider an acute psychosis with incidental positive enterovirus PCR. Because of the association of headache and hallucinations, migraine could also be considered as the cause of the symptoms. However, the time course and presentation of symptoms both speak against this hypothesis.

To our knowledge, we are the first to report a case of isolated visual and auditory hallucinations caused by an enterovirus encephalitis. In our case, the content of the visual and auditory hallucinations were not trivial but with a strong imperative character telling the patient to commit suicide. Notably, there was a chronological order of the occurrence of diarrhoea, auditory, and visual hallucinations. PCR testing for enteroviruses in CSF should be considered when there is an acute onset of new neuropsychiatric symptoms. and an enterovirus infection is suspected (e.g., severe diarrhea in the past days/week).

## Data Availability

The data are not publicly available due to privacy or ethical restrictions.
